# A case of bony injury and osteomyelitis during the Covid-19 lockdown

**DOI:** 10.6026/973206300213956

**Published:** 2025-10-31

**Authors:** Sobiya Farook, Alina Sanda Gilligan

**Affiliations:** 1 Department of Medicine and Education, East Lancashire Hospitals NHS Trust, Blackburn, UK; 2 Department of Geriatric Medicine, East Lancashire Hospitals NHS Trust, Blackburn, UK

**Keywords:** Leg ulcers, osteomyelitis, self-neglect, cognitive impairment, social isolation, Covid19

## Abstract

Social isolation has been linked with psychological and cognitive decline and this has become more pertinent during the Covid19 pandemic. Here we report a case of self-inflicted tibial bony defect with associated osteomyelitis due to leg ulcers, self-neglect, and lack of support during self-isolation relating to Covid-19 restrictions. The soft tissue and bony defect created was extensive and it is likely caused by a support stocking acting as a tourniquet over a long time. The patient developed osteomyelitis and septicaemia and was treated with IV antibiotics. She was found to have mild cognitive impairment and was referred to community memory services. Thus, successive lockdowns with subsequent self-elected isolation have serious consequences for physical and mental well-being.

## Case report:

An 82-year-old female was admitted with pyrexia, left leg ulcers and decreased mobility. Past medical history included hypertension, gallstones, breast cancer and previous leg ulcers. Examination identified a deep circumferential transversal ulcer with associated tissue loss just above the left ankle, which anteriorly extended to the tibia. The ulcer was wet and sloughy with malodour [1]. The leg above was severely swollen, with several broken skin blisters. The left heel also had a grade 4 pressure ulcer. Peripheral pulses were present. There was paraesthesia of the left heel area and loss of pinprick sensation on the medial dorsal aspect of the left foot, including the hallux [2]. The patient had Staphylococcal septicaemia and a swab culture grew Staph Aureus and Strep G. An x-ray showed a deep bony defect within the distal tibia and fibula with periosteal reaction along the medial border of the tibia. Given the straight borders, the defect looked iatrogenic [3]. This was however inconsistent with the history. An MRI confirmed osteomyelitis. A six-week course of intravenous antibiotics (Ceftriaxone) was commenced. After two weeks, she could be discharged to a rehabilitation facility to complete the antibiotics and for further physiotherapy input [4]. The patient lived alone and had become increasingly frail and with reduced mobility over the preceding year. Her family had growing concerns regarding personal care, however the patient declined support. She had started isolating before the start of the official Covid-19 lockdown [5] and remained isolated for a year. She expressed statements suggesting an abnormal level of anxiety regarding possible Covid-19 transmission. Concerns were raised by delivery drivers, regarding suspicion of self-neglect and poor personal hygiene [6]. She denied this when the GP telephoned. She was judged to have capacity regarding personal care and given the higher threshold for home consultations during lockdown, no home visits were conducted. The patient called the out-of-hours services shortly prior to admission [7]. She was struggling to remove her stocking and "an old ulcer had started bleeding". This resulted in three visits from the district nurses to dress the leg before she became septic and collapsed at home, necessitating an emergency admission. It was challenging to get a good history from the patient, even once she was feeling better. Based on conversations with the patient, her family and district nurse evidence, we believe a support knee sock acted as a tourniquet above the ankle, as it was no longer possible for the patient to pull it up the leg. This caused an increasingly deeper injury into the tissues, including the bone. Insight into the severity of her injury remained poor and she scored 68/100 in an ACE-III cognitive test. She had no history of cognitive deficits and was referred to memory services on discharge.

## Discussion:

This case of severe self-neglect and isolation was triggered by the Covid-19 pandemic. To the best of our knowledge, no previous case of injury stemming from poor care progressed to this stage. Implications of Covid-19 restrictions in a susceptible individual meant abnormal levels of anxiety, isolation and breakdown of social networks [8]. The modified primary care response during the pandemic led to prolonged uninformed self-care [9]. Continued isolation contributed to increasing frailty, deterioration in cognition and delusional thoughts and beliefs. Several studies, including a recent meta-analysis, have linked social isolation with cognitive deterioration and increased risk of dementia [10]. This case shows the potential negative impact on health of social isolation, in this instance leading to severe bony injury with associated osteomyelitis. Consideration of the risks that isolation poses to the physical and mental health of vulnerable population groups should be taken into consideration when planning new Covid19 restrictions, if there is an outbreak in the future.

## Consent for publication:

Written informed consent was obtained from the patient for publication of her clinical details and radiograph.
